# Determination of the Phylogenetic Relationship of *Dendrobium linawianum* (Orchidaceae) Based on Comparative Analysis of Complete Chloroplast Genomes

**DOI:** 10.3390/cimb47100869

**Published:** 2025-10-21

**Authors:** Fengping Zhang, Qiyong Huang, Yaqiong Zhang, Dongqin Lǚ, Rui Chen, Yanshu Jia, Qiongchao Li

**Affiliations:** College of Ethnic Medicine, Yunnan Key Laboratory of Dai and Yi Medicines, Yunnan International Joint Laboratory of China-Laos Traditional Medicine, Yunnan University of Chinese Medicine, Kunming 650500, China

**Keywords:** identification, chloroplast genomic sequence, *Dendrobium linawianum*, genomic comparison, Orchidaceae

## Abstract

*Dendrobium* is an orchid genus with high economic and ecological importance, but its taxonomy based on morphology remains controversial. *Dendrobium linawianum*, a critically endangered species with both ornamental and medicinal value, represents a key taxon within this genus. However, its phylogenetic relationship has long been unplaced due to similar morphological traits. Despite its conservation and taxonomic importance, its complete chloroplast genome has not been previously characterized. Here, we newly sequenced and assembled the complete chloroplast genome of *D*. *linawianum*. The 150,497 bp genome exhibits a typical quadripartite structure, encoding 119 genes. A total of 161 simple sequence repeats (SSRs) were identified, predominantly mononucleotide and dinucleotide motifs. Condon usage analysis revealed leucine as the most abundant amino acid. Phylogenetic analysis based on complete chloroplast genome sequences strongly supported the close relationship of *D*. *linawianum* with *D*. *hercoglossum*, *D*. *thyrsiflorum*, and *D*. *moniliforme*, resolving its taxonomic position within the genus. The complete chloroplast genomes successfully resolved the phylogenetic relationships among 35 *Dendrobium* species, demonstrating their efficacy as powerful molecular markers for resolving taxonomic ambiguities within this morphologically complex genus. Our findings provide a genomic foundation for precise species identification and molecular breeding of *D*. *linawianum*, and enhance understanding of phylogenetic relationships in this taxonomically challenging group.

## 1. Introduction

*Dendrobium* Sw., one of the largest genera in the Orchidaceae family, comprises over 1500 species distributed across tropical and subtropical regions of Asia, Australia, and the Pacific Islands [1,2,3]. For centuries, numerous *Dendrobium* species have been highly valued in Asian traditional medicine and horticulture [4,5,6,7]. Owing to population declines driven by overharvesting and habitat loss, many *Dendrobium* species are listed under the Convention on International Trade in Endangered Species (CITES) [8]. However, the high commercial value of these orchids has resulted in frequent species adulteration in commercial markets [9,10]. Consequently, developing reliable species identification methods is crucial for enabling effective conservation of endangered populations, promoting the sustainable utilization of *Dendrobium* genetic resources, and facilitating the targeted development of new horticultural hybrids within this genus. Notably, the taxonomy of *Dendrobium* is widely regarded as one of the most complex challenges in Orchidaceae, largely due to vegetative similarity among closely related species [11,12,13]. Traditional classification relied primarily on morphological characteristics [5,14,15,16,17,18,19], with supplementary support from analytical techniques including capillary electrophoresis [20] and high-performance liquid chromatography [21]. However, these methods are often insufficient for resolving closely related species within specific clades, such as the one containing *D*. *linawianum*. This species exhibits high morphological similarity and overlapping morphological character states (such as stem shape and leaf number) with *D*. *nobile*, leading to persistent uncertainties in its delineation and classification [22]. This ambiguity directly underscores the necessity of employing more powerful molecular tools for achieving a reliable and definitive phylogenetic resolution [1,23].

Recent advances in molecular techniques have significantly improved the accuracy and efficiency of *Dendrobium* species identification [7,24,25,26]. Among these techniques, DNA barcoding, whether utilizing single or combined markers, has proven particularly valuable for clarifying interspecific relationships [1,22,27]. Nevertheless, phylogenetic uncertainties persist for certain closely related species within the genus [1,28]. This gap highlights the need for more informative molecular tools to achieve robust species discrimination. Chloroplast genomes (cpDNA) have emerged as powerful alternatives for phylogenetic reconstruction and species authentication in *Dendrobum* [29,30,31]. As maternally inherited, semi-autonomous genetic units, chloroplast genomes exhibit high conservation in gene content and syntenic arrangements across most angiosperms [32], typically featuring a canonical quadripartite structure: two inverted repeat (IR) regions flanking a large single-copy (LSC) region and a small single-copy (SSC) region [33]. Compared to nuclear genomes, chloroplast genomes have lower base substitution rates and fewer genomic rearrangements, which ensure stable phylogenetic signals while still retaining sufficient variation to distinguish closely related species [29,31,34]. These characteristics make chloroplast DNA ideal for resolving the taxonomic complexities and phylogenetic uncertainties that plague *Dendrobium* classification.

**Figure 1:**
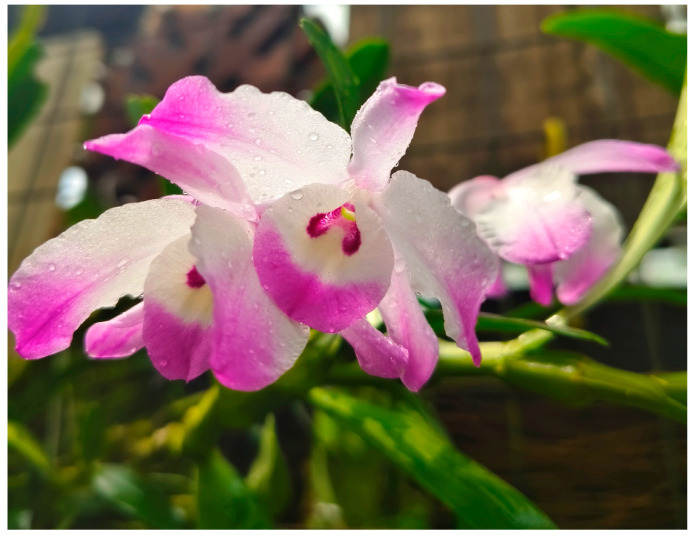
The flowering plant of *Dendrobium linawianum*.

*Dendrobium linawianum* Rchb. f. is a commercially and ecologically valuable orchid, prized both for its ornamental floral traits and its medicinal potential [35,36] (Figure 1). However, the complete chloroplast genome of *D. linawianum*, a key resource for genetic characterization and phylogenetic analysis, remains unreported. This knowledge gap has hindered comprehensive efforts to clarify its genomic features, guide germplasm preservation, and resolve its evolutionary placement within the *Dendrobium* genus. To address these limitations, we used Illumina Hiseq high-throughput sequencing to generate chloroplast genome data for *D. linawianum*, followed by de novo assembly and detailed annotation to construct a complete chloroplast genome map. The specific objectives of this study were: (1) to characterize the structural features of *D. linawianum* chloroplast genome and compare it with closely related *Dendrobium* species; and (2) to reconstruct the phylogenetic position of *D. linawianum* within *Dendrobium* using complete chloroplast genome sequences. Our findings provide foundational genomic data for *D. linawianum*, which will not only facilitate accurate species identification but also advance broader phylogenetic and conservation genetic studies of the taxonomically complex *Dendrobium* genus.

## 2. Materials and Methods

### 2.1. Collection of Leaf Sample from Dendrobium linawianum and DNA Sequencing

Fresh, healthy leaves of *D. linawianum* were collected from the Kunming Institute of Botany, Chinese Academy of Sciences, leaf samples were immediately flash-frozen in liquid nitrogen, and subsequently stored at −80 °C until DNA extraction. Voucher specimens were deposited in the Herbarium of the Kunming Institute of Botany, Chinese Academy of Sciences. Genomic DNA was extracted using a modified cetyltrimethylammonium bromide (CTAB) method [37]. High-quality DNA samples were sequenced on an Illumina NovaSeq 6000 platform (Illumina, San Diego, CA, USA).

### 2.2. Chloroplast Genome Assembly and Annotation

Clean data were filtered using GetOrganelle [38] were subsequently assembled into a complete chloroplast genome using SPAdes v.3.11.1 [39]. Genome annotation was performed using CpGAVAS2 [40]. The assembled chloroplast genome had an average coverage depth of 2741.92× with 100% genome coverage (Figure S1). A circular map of the *D*. *linawianum* chloroplast genome was visualized using the Organellar Genome DRAW [41]. The final annotated chloroplast genome sequence of *D*. *linawianum* has been deposited in GenBank under accession number NC087858.

### 2.3. Repeat and Codon Usage Analyses, Genome Comparison

Simple sequence repeats (SSRs) in the *D. linawianum* chloroplast genome were identified using MISA v2.1 [42] with minimum repeat thresholds set to 8, 5, 4, 3, 3, and 3 for mono-, di-, tri-, tetra-, penta-, and hexa-nucleotides, respectively. Codon preference was analyzed using CodonW v1.4.4 [43] to calculate relative synonymous codon usage (RSCU). The boundaries of inverted repeats (IRs), small single-copy (SSC), and large single-copy (LSC) regions were compared among *D. linawianum*, *D. strongylanthum*, *D. nobile*, *D. thyrsiflorum*, *D. parishii*, *D. brymerianum*, *D. chrysotoxum*, *D. jenkinsii*, *D. moniliforme*, and *D. strongylanthum* using the online tool IRscope [44] (https://irscope.shinyapps.io/irapp/) (accessed on 4 November 2024).

### 2.4. Phylogenetic Analysis

Phylogenetic analysis was performed using complete chloroplast genome sequences from 37 species, with *Phalaenopsis equestris* and *P. Aphrodite* designated as outgroups. The sequence of *D. linawianum* was newly generated in this study, while chloroplast genomes of the remaining 34 *Dendrobium* species and the two outgroup taxa were obtained from the National Center for Biotechnology Information (NCBI) GenBank database. All chloroplast genome sequence was aligned with MAFFT v7.307 [45]. Phylogenetic relationships were reconstructed using Bayesian inference (BI) and Neighbor-Joining (NJ) methods. The BI analysis was performed using MrBayes v3.2 [46] with four Markov chains running for 10 million generations. The NJ tree was constructed in MEGA11 [47] with 1000 rapid bootstrap replicates.

## 3. Results

### 3.1. Genomic Features of Dendrobium linawianum Complete Chloroplast Genome

**Figure 2:**
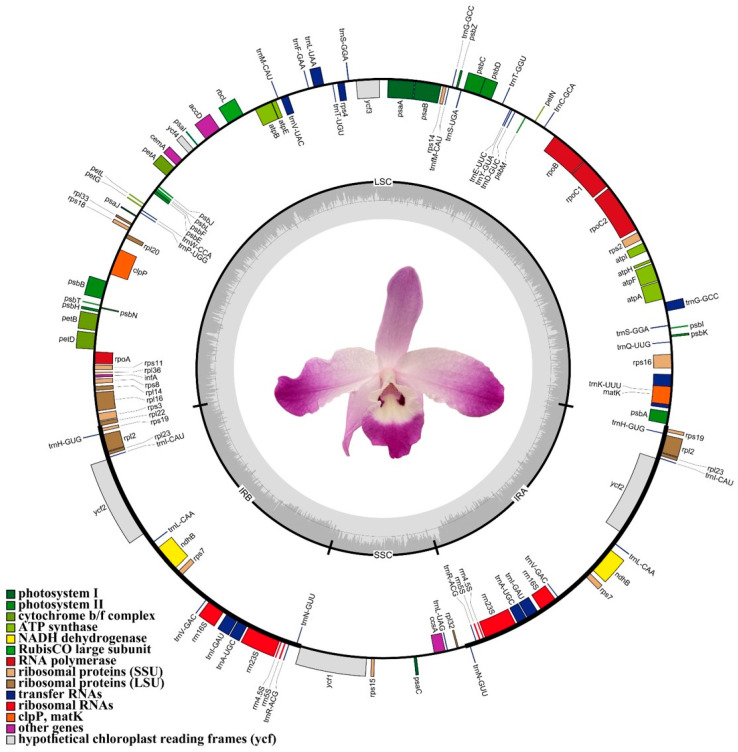
Gene maps of the complete chloroplast genome of *Dendrobium linawianum*. Genes on the inside of the circle are transcribed clockwise, while those on the outside are transcribed counterclockwise. The same gene type is represented by the same color.

The complete chloroplast genome of *D*. *linawianum* was sequenced and assembled. The genome is 150,497 bp in length and exhibits the typical structural features of angiosperm chloroplast genomes: a circular, double-stranded DNA with a quadripartite structure. Specifically, it consists of two Inverted Repeats regions (IRs) (25,970 bp each), a Large Single-Copy region (LSC) (84,770 bp), and a Small Single-Copy region (SSC) (13,787 bp) (Table 1; Figure 2). The total GC content of the *D*. *linawianum* chloroplast genome is 37.56%, corresponding to an AT content of 62.44%, indicating a clear AT bias. GC content varies across the four genomic regions: the highest GC content is observed in the IRs (43.42%), followed by the LSC (35.13%) and the SSC region (30.48%) (Table 1).

The complete chloroplast genome of *D*. *linawianum* was annotated to contain 119 genes, consisting of 74 protein-coding genes, 37 tRNA genes, and 8 rRNA genes. These genes were functionally classified into four categories: (1) photosynthesis-related genes, (2) self-replication genes, (3) other genes, and (4) genes with unknown functions. Six protein-coding genes (*rps19*, *rpl2*, *rpl 23*, *ycf2*, *ndhB*, *rps7*), eight tRNA genes (*trnH-GUG*, *trnI-CAU*, *trnL-CAA*, *trnV-GAC*, *trnI-GAU*, *trnA-UGC*, *trnR-ACG*, *trnN-GUU*), and all four rRNA genes (*rrn5S*, *rrn4.5S*, *rrn23S*, and *rrn16S*) are located in the IRs region. Chloroplast genome annotation revealed that 16 genes contain introns: two genes (*clpP*, *ycf3*) contain two introns, while the remaining 14 genes have a single intron (Table 2).

The chloroplast genome of *D. linawianum* encoded 37 tRNAs, 20 of which are duplicated. These duplicated genes include 10 tRNAs (*trnS-GGA*, *trnG-GCC*, *trnH-GUG*, *trnI-CAU*, *trnL-CAA*, *trnV-GAC*, *trnI-GAU*, *trnA-UGC*, *trnR-ACG*, *trnN-GUU*), all 4 rRNAs (*rrn5S*, *rrn4.5S*, *rrn23S*, *rrn16S*), and 6 protein-coding genes (*rps19*, *rps12*, *rpl23*, *ycf2*, *ndhB*, *rps7*) (Table 2). Among all intron-containing genes in the *D. linawianum* chloroplast genome, the *trnK-UUU* gene contained the largest intron (2781 bp). Analysis of start codons revealed that genes *rps16*, *atpF*, *rpoC1*, *ycf3*, *clpP*, *petB*, *petD*, *rp116*, and *dhB* used ATG as a start codon, *rp12* initiated with ATA, while *trnK*-*UUU*, *trnL*-*UAA*, *trnI*-*GAU*, and *trnA*-*UGC* began with GGG (Table 3). The chloroplast genome of *D. linawianum* had 16 intron-containing genes, among which 14 (eight protein-coding genes and six tRNA genes) had a single intron, and two genes (*ycf3* and *clpP*) had two introns each. Twelve genes (eight protein-coding and four tRNA genes) were located in the LSC region, and four genes (two protein-coding and two tRNA genes) in the IR regions. The pattern of intron presence was a common feature of a variety of genes in the chloroplast genomes of the Orchidaceae.

### 3.2. Repeat Sequence and Codon Usage Analyses of Complete Chloroplast Genome in D. linawianum

**Figure 3:**
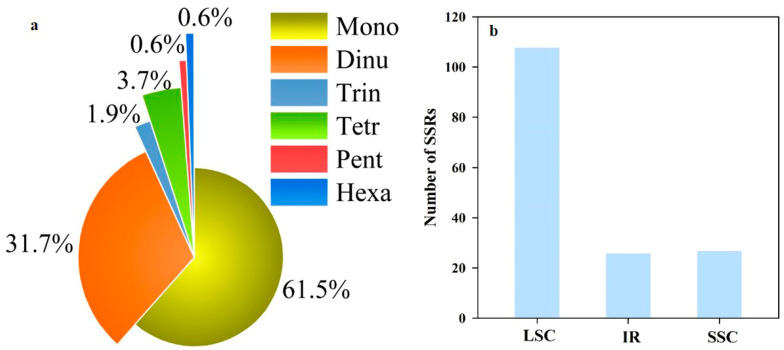
Distribution of SSRs (simple sequence repeat) in the *Dendrobium linawianum* chloroplast (CP) genomes. (**a**) SSR type and the proportion in the *Dendrobium linawianum* CP genomes; (**b**) The number of SSRs in different genomic regions of *Dendrobium linawianum* CP genomes. Mono, mononucleotide SSRs; dinu, dinucleotide SSRs; trin, trinucleotide SSRs; tetr, tetranucleotide SSRs; pent, pentanucleotide SSRs; hexa, hexanucleotide SSRs; LSC, large single-copy region; SSC, small single-copy region; IR, inverted repeat region.

A total of 161 SSRs were identified in the *D. linawianum* chloroplast genome, comprising 99 mononucleotides (61.49%), 51 dinucleotides (31.68%), 3 trinucleotides (1.86%), 6 tetranucleotides (3.73%), 1 pentanucleotide (0.62%), and 1 hexanucleotide (0.62%) (Figure 3a). This dominance of mono- and di-nucleotide repeats is consistent with the AT-rich compositional bias of *D. linawianum* chloroplast genomes. Distribution analysis further revealed 108 SSRs (67.08%) in the LSC region, 26 (16.15%) in the IRs, and 27 (16.77%) in the SSC (Figure 3b).

**Figure 4:**
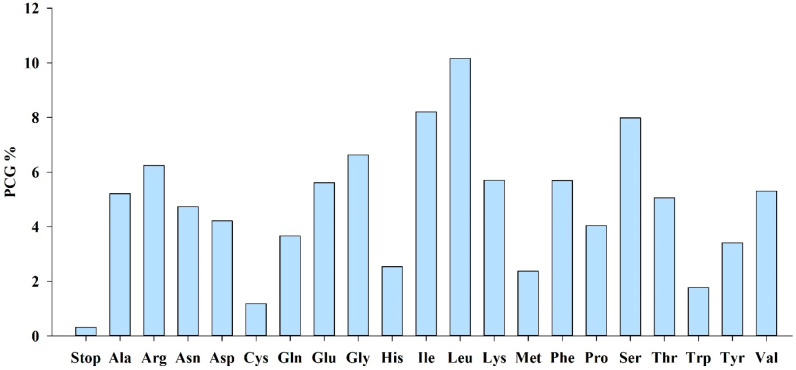
Codon distribution in the chloroplast genome of *Dendrobium linawianum*. PCG, Protein-coding gene.

The chloroplast genome of *D. linawianum* contains 43,456 protein-coding codons, encoding 20 amino acids with variation in frequency. Leucine (Leu) was the most frequently encoded amino acid (4381 codons, 10.08%), while cysteine (Cys) was the least frequent with only 530 codons (1.22%) (Figure 4; Table 4). In terms of synonymous codon diversity, tryptophan (Trp, encoded exclusively by UGG) and methionine (Met, encoded exclusively by AUG) were the only amino acids specified by a single codon. In contrast, leucine (Leu), arginine (Arg), and serine (Ser) each utilized six synonymous codons, while alanine (Ala), glycine (Gly), proline (Pro), threonine (Thr), and valine (Val) each employed four. The remaining amino acids (Cys, Asp, Glu, Phe, His, Lys, Asn, Gln, Tyr) use two synonymous codons. Relative synonymous codon usage (RSCU) analysis of 64 total codons revealed a strong AT-biased preference: 32 codons had RSCU values >1, of which 29 ended in A or U and only three ended in G or C. Among these, the arginine-encoding codon AGA was the most frequently used (RSCU = 1.88), while the arginine-encoding codon CGC was the least preferred (RSCU = 0.33) (Table 4).

### 3.3. IR/SC Boundary Analysis of Chloroplast DNA in D. linawianum

**Figure 5:**
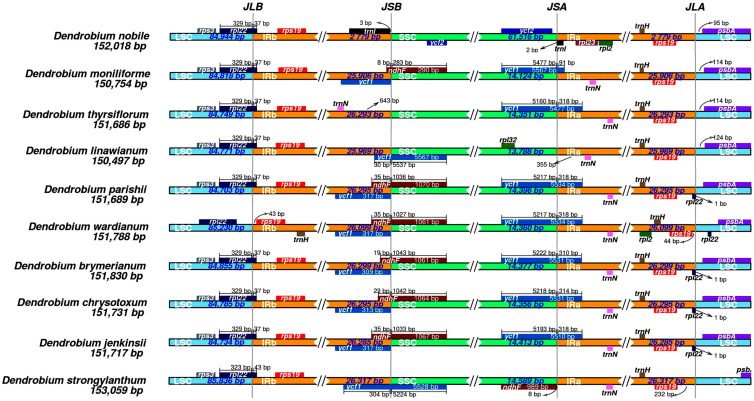
Comparison of the regions flanking inverted repeat/single copy (IR/SC) junctions among ten *Dendrobium* species.

The chloroplast genome of *Dendrobium* species was analyzed by examining the SC/IR boundaries of chloroplast gene sequences of 10 species of *Dendrobium* species from different branches, which presented four boundaries, LSC-IRb, SSC-IRb, SSC-IRa, and LSC-IRa, respectively. Among them, the LSC-IRb boundary is relatively conservative, mostly located within the coding region of the *rpl22* gene. The boundaries of SSC-IRb largely vary. *D*. *linawianum and D*. *strongylanthum* miss the *ndhF* gene on the right side, *D*. *nobile* and *D*. *thyrsiflorum* miss both *ycf1* and *ndhF* genes, the boundaries of SSC-IRb of the other six *Dendrobium* species are located in the overlapping region of *ycf1* and *ndhF* genes, and extend into the coding region of the *ndhF* gene. The boundaries of SSC-IRa are located on the inside of the *ycf1* gene, only *D*. *linawianum* miss the *ycf1* gene. The boundaries of LSC-IRa of *D*. *parishii*, *D*. *brymerianum*, *D*. *chrysotoxum*, and *D*. *jenkinsii* were located in the non-coding region between the *rpl22* and *psbA* genes, and tend to be closer to the *rpl22* gene, while the boundaries of LSC-IRa of *D*. *nobile*, *D*. *moniliforme*, *D*. *thyrsiflorum*, and *D*. *strongylanthm* lack the *rpl22* gene. Overall, among the four boundaries of the complete chloroplast gene sequence in *Dendrobium* species, the SSC-IR boundary exhibits significant variation, while the LSC-IR boundary is relatively conservative (Figure 5).

### 3.4. Characteristics of the Genus Dendrobium Chloroplast Genome

**Figure 6:**
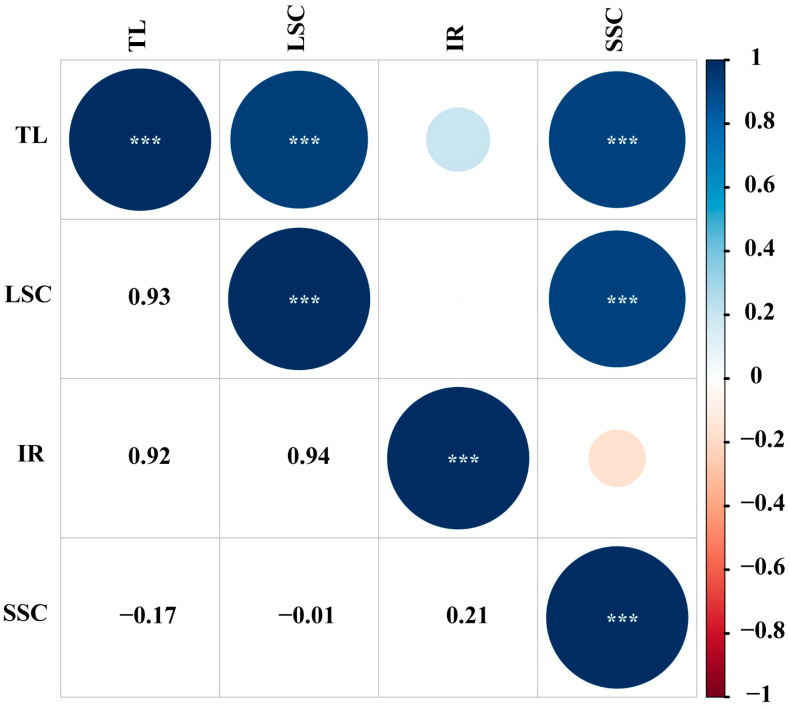
Pearson’s correlations among the sizes of whole genome, large single-copy, small single-copy, and inverted Repeats for the 34 studied *Dendrobium* species. Circle sizes and colors represent the significance and correlation coefficient (*r*). Significant levels are shown. *** *p* < 0.001. TL, total length of whole genome; LSC, large single-copy region; SSC, small single-copy region; IR, inverted repeat region.

The complete chloroplast genome lengths of the 35 analyzed *Dendrobium* species ranged from 150,497 bp (*D*. *linawianum*) to 160,024 bp (*D*. *longicornu*) (Table S1). The chloroplast genome of *D*. *linawianum* was relatively smaller compared to the chloroplast genomes of other *Dendrobium* species. Furthermore, the size of the complete chloroplast genome from the studied *Dendrobium* species was positively significantly correlated with the sizes of the large single-copy region (LSC) and small single-copy region (SSC), but not correlated with inverted repeat regions (IRs) (Figure 6). The AT content of 35 *Dendrobium* chloroplast genomes was highly conserved, ranging only from 62.3% (*D*. *strongylanthum*) to 62.9% (*D*. *cariniferum* and *D. longicornu*) (Table S1).

### 3.5. Phylogenetic Relationships

**Figure 7:**
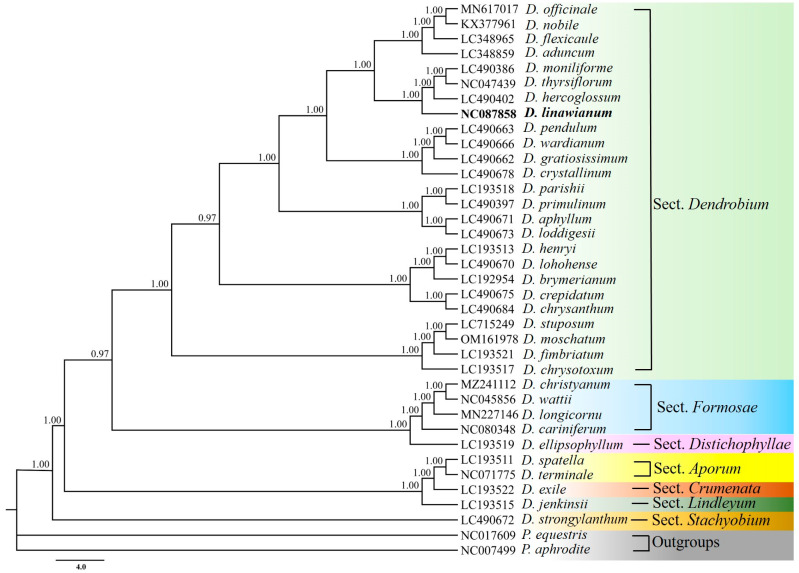
Bayesian inference (BI) phylogeny of *Dendrobium linawianum* and other 34 *Dendrobium* species based on chloroplast genomes.

**Figure 8:**
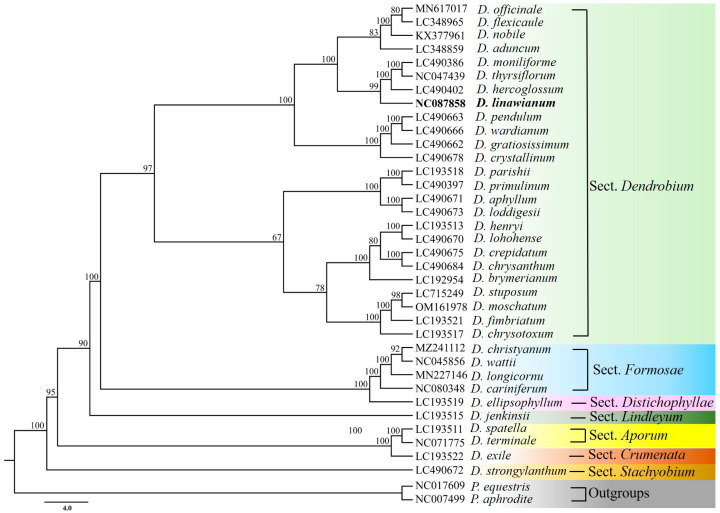
Neighbor-Joining (NJ) phylogeny of *Dendrobium linawianum* and other 34 *Dendrobium* species based on the complete chloroplast genome sequences. Numbers above the branches indicate bootstrap percentages (BS).

Phylogenetic relationships among *D. linawianum* and 34 other *Dendrobium* species were referred using complete chloroplast genome sequences. All branch nodes received strong support in both Bayesian inference (BI) (Figure 7) and Neighbor-Joining (NJ) (Figure 8) analyses. In both phylogenetic trees, all the sampled *Dendrobium* species formed a well-supported monophyletic clade. *D. linawianum* was most closely related to *D. hercoglossum*, with strong support from both analytical methods: a posterior probability (PP) of 1.00 in the BI tree and a bootstrap value (BS) of 100% in the NJ tree.

## 4. Discussion

The complete chloroplast genome of *Dendrobium linawianum*, a critically endangered orchid of ecological and medicinal significance provides critical insights into its genomic architecture, evolutionary relationships, and conservation-oriented breeding. This study reveals that *D. linawianum* chloroplast genome conforms to the canonical structural pattern of most angiosperms, featuring a quadripartite organization (LSC, SSC, and two IR regions) and conserved gene content [30,31], it also exhibits distinct characteristics that reflect both its adaptive strategies and its phylogenetic placement within the genus *Dendrobium*. These findings not only enrich our understanding of *Dendrobium* chloroplast genome evolution but also lay a foundation for targeted conservation efforts for this endangered species.

The chloroplast genome of *D. linawianum* adheres to the canonical quadripartite structure characteristic of land plant chloroplasts, comprising two identical inverted repeat (IR) regions, one large single-copy (LSC) region, and one small single-copy (SSC) region [48]. With a total length of 150,497 bp, it is among the smaller chloroplast genomes in *Dendrobium*, where genome sizes range from 150,497 bp (*D. linawianum*) to 160,024 bp (*D. longicornu*). This interspecific size variation aligns with broader trends observed in angiosperm chloroplast genomes: total genome length is primarily shaped by expansions or contractions of the LSC and SSC regions, while the IR regions remain relatively stable [49,50,51]. The correlation analysis further supports this pattern: we detected a significant positive correlation between total chloroplast genome size and the lengths of both the LSC and SSC regions, but no significant association with IR length. This suggests that *Dendrobium* chloroplast genome evolution is driven predominantly by size changes in the single-copy regions, possibly driven by insertions, deletions, or gene rearrangements.

The GC content of *D. linawianum* chloroplast genome (37.56%) and its regional distribution pattern across regions (IRs > LSC > SSC), which is consistent with that of other *Dendrobium* species and most Orchidaceae taxa [30,31]. The higher GC content in IRs (43.42%) likely reflects the presence of rRNA and tRNA genes, which are GC-rich to stabilize secondary structures critical for translation [32]. The overall AT bias (62.44%) of the *D. linawianum* is a common feature of chloroplast genomes, attributed to the uniparental inheritance and reduced recombination of chloroplast DNA, which limits the correction of AT-enriched mutations [32,52]. The gene content of the *D. linawianum* chloroplast genome encodes 119 unique genes, including 74 protein-coding genes, 37 tRNAs, and 8 rRNAs. This gene set closely mirrors that of closely related *Dendrobium* species [29,30,53]. Functional categorization of these genes into photosynthesis-related, self-replication, and other roles highlights the chloroplast’s dual function as a site of photosynthetic energy production and a semi-autonomous genetic unit capable of self-maintenance. The six protein-coding genes, ten tRNAs, and four rRNAs are duplicated in the IRs, a phenomenon thought to protect these essential genes from deleterious mutations through recombination-dependent repair [54].

Simple sequence repeats (SSRs) in chloroplast genomes are widely recognized as valuable molecular markers for studying genetic diversity and population structure due to their high polymorphism [55,56,57,58]. In the *D*. *linawianum* chloroplast genome, we identified 161 SSRs, with a clear dominance of mono- and di-nucleotide repeat motifs, accounting for 93.17% of all detected SSRs. This motif distribution exhibits a strong AT bias, which aligns with the overall AT-rich composition of the *D*. *linawianum* chloroplast genome. Most SSRs are localized in the LSC region (67.08%), which is known to accumulate more mutations than the IR regions due to lower selective constraint [59]. These SSRs can serve as species-specific markers for monitoring wild populations of *D. linawianum*, aiding in the detection of illegal trade and supporting ex situ conservation efforts. We propose two key research directions. First, the development of a standardized molecular identification toolkit based on a panel of highly polymorphic SSRs is essential for the rapid and accurate authentication of *D. linawianum*, which is crucial for curbing illegal trade. Second, a comprehensive population genetics study utilizing these markers should be undertaken to assess the genetic diversity and structure of remaining wild populations. Understanding the distribution of genetic variation will reveal potential inbreeding depression, identify genetically unique populations, and provide a scientific basis for prioritizing conservation units and designing effective breeding programs in germplasm banks.”

Codon usage analysis of *D*. *linawianum* chloroplast genome reveals that leucine was the most abundant amino acid (10.08%), while cysteine was the least (1.22%). This amino acid abundance pattern is highly conserved, not only observed in other *Dendrobium* species [30,31], but also across angiosperm chloroplast genomes more broadly. The strong AT bias in codon usage 29 of 32 preferred codons end in A/U) likely reflects the chloroplast genome’s AT-rich composition and may enhance translation efficiency by matching the tRNA pool [60]. Variation, contraction, and expansion of inverted repeat (IR) regions represent common evolutionary phenomena in flowering plants [61]. These structural changes frequently occur at IR-single copy region junctions (LSC/IRa, IRb/SSC), resulting in boundary shifts that relocate genes between IR and single-copy regions [62]. Furthermore, *ndhF* genes were absent in *D. linawianum*, *D. nobile*, *D. thyrsiflorum*, and *D. strongylanthum*. Such chloroplast *ndh* gene losses typically result from functional degradation or nuclear genome transfer [63,64], with fungal symbiosis potentially contributing to this phenomenon as observed in other orchids [65]

Comparative analysis of the *D. linawianum* chloroplast genome with those of 34 *Dendrobium* species confirms that *D. linawianum* has a relatively small genome, a trait that may reflect adaptive evolution to its ecological niche. Smaller chloroplast genomes are often associated with increased energy efficiency or reduced genetic load [66,67], which could be advantageous for *D. linawianum* in its native montane forest habitats, where resources are limited [5,6]. We speculate that this genomic streamlining might have a complex relationship with the species’ current endangered status [68]. Historically, a more compact, energetically efficient genome could have been beneficial for survival in stable but resource-limited niches. However, this potential specialization might come at a cost of reduced genetic plasticity. In the face of rapid environmental changes, such as habitat fragmentation and climate change, a smaller, highly conserved genome could potentially harbor less adaptive variation, thereby limiting the species’ ability to respond to new stressors and increasing its vulnerability to extinction. The conserved AT content (62.3–62.9%) across *Dendrobium* further supports the genus’s genetic cohesion, despite its morphological diversity.

Accurate identification of medicinal plants is essential for safe utilization, hybrid breeding, and genetic resource conservation. However, *Dendrobium* species are notoriously challenging to discriminate morphologically due to high phenotypic similarities among closely related taxa, particularly during non-flowering stages [11,12,13]. In recent years, chloroplast genomes, with their abundant variable sites, have emerged as powerful tools for phylogenetic reconstruction and species authentication in taxonomically complex plant genera including *Orychophragmus*, *Cymbidium*, *Schima*, and *Dendrobium* [69,70,71,72,73,74,75]. In this study, complete chloroplast genomes were used to authenticate *D. linawianum* and resolve its phylogenetic relationships with closely related *Dendrobium* species. Phylogenetic analysis based on 35 *Dendrobium* chloroplast genomes robustly resolved *D. linawianum* as sister to *D. hercoglossum* (PP = 1.00, BS = 100), clarified relationships among morphologically *D. linawianum* and *D*. *nobile*, which were previously unplaced species based on stem shape and leaf number alone [22]. This resolution highlights the utility of complete chloroplast genomes in overcoming taxonomic challenges posed by convergent morphology and hybridization in *Dendrobium* [73].

Future studies that integrate whole nuclear genome data will be crucial for investigating several key processes. Specifically, nuclear genomes can reveal histories of hybridization and introgression events by detecting discordant gene trees and introgressed alleles, which are invisible to chloroplast data. They are also essential for deciphering the role of polyploidization, a common phenomenon in orchids, in the evolution of this genus. Furthermore, a combined analysis of nuclear and chloroplast markers would provide a more holistic view of genetic diversity, allowing us to assess whether patterns of cytoplasmic (chloroplast) diversity align with those of nuclear diversity, and to identify genomic regions under selection that may be linked to local adaptation and the species’ critically endangered status.

## 5. Conclusions

Comparative chloroplast genomic analysis of *Dendrobium linawianum* with 34 congeneric species sheds light on the evolution of *Dendrobium* chloroplast genomes. Our findings demonstrate that *D. linawianum* exhibits highly conserved genome content and arrangement, which are consistent with other *Dendrobium* species. Its chloroplast genome size is shaped primarily by LSC and SSC lengths. Phylogenetic analyses using complete chloroplast genomes confirm its close affinity to other *Dendrobium* species, particularly *D. hercoglossum*. The *D. linawianum* chloroplast genome provides a robust reference for resolving phylogenetic ambiguities in *Dendrobium* and offers practical tools for species authentication. The genomic resources generated in this study, including complete *D. linawianum* chloroplast sequences, SSR markers, and phylogenetic insights, support both basic research on orchid evolution and applied efforts to conserve and improve ornamental and medicinal *Dendrobium* germplasm. Integrating chloroplast and nuclear genomic approaches in future studies will further clarify the evolutionary processes shaping this ecologically and economically important genus.

## Figures and Tables

**Table 1:** General features of *Dendrobium linawianum* chloroplast genome.

Region	Size (bp)	T%	C%	A%	G%	A+T%	G+C%
Whole genome	150,497	31.45	18.94	30.99	18.62	62.44	37.56
LSC	84,771	33.18	17.95	31.69	17.18	64.87	35.13
SSC	13,788	32.7	14.59	36.82	15.89	69.52	30.48
IRA	25,969	28.28	20.96	28.3	22.46	56.58	43.42
IRB	25,969	28.28	20.96	28.3	22.46	56.58	43.42

Notes: LSC, Large Single-Copy; SSC, Small Single-Copy; IRA, Inverted Repeat A; IRB, Inverted Repeat B.

**Table 2:** Annotation of genes of the chloroplast genome in *Dendrobium linawianum*.

Types of Genes	Group of Genes	Name of Genes
	Photosystem I gene	*psaA*, *psaB*, *psaC*, *psaI*, *psaJ*
	Photosystem II gene	*psbA*, *psbB*, *psbC*, *psbD, psbE*, *psbF*, *psbH*, *psbI*, *psbJ*, *psbK*, *psbL*, *psbM*, *psbN*, *psbT*, *psbZ*
Photosynthesis	Cytochrome b/f complex gene	*petA*, *petB* *, *petD* *, *petG*, *petL*, *petN*
	ATP synthase gene	*atpA*, *atpB*, *atpE*, *atpF* *, *atpH*, *atpI*
	Rubisco gene	rbcL
	NADH-dehydrogenase gene	*ndhB* ^a^*
Self-replication genes	RNA polymerase gene	*rpoA*, *rpoB*, *rpoC1* *, *rpoC2*
	Large subunit of the ribosome	*rpl14*, *rpl16* *, *rpl2* ^a^*, *rpl20*, *rpl22*, *rpl23* ^a^, *rpl32*, *rpl33*, *rpl36*
	Small subunit of the ribosome	*rps11*, *rps14*, *rps15*, *rps16* *, *rps18*, *rps19* ^a^, *rps2*, *rps3*, *rps4*, *rps7* ^a^, *rps8*
	Ribosomal RNAs gene	*rrn4.5S* ^a^, *rrn5S* ^a^, *rrn16S* ^a^, *rrn23S* ^a^
	Transfer RNAs gene	*trnK-UUU* *, *trnQ-UUG*, *trnG-GCC* ^a^*, *trnC-GCA*, *trnD-GUC*, *trnY-GUA*, *trnE-UUC*, *trnT-GGU*, *trnS-UGA*, *trnS-GGA* ^a^, *trnT-UGU*, *trnL-UAA* *, *trnF-GAA*, *trnV-UAC* *, *trnW-CCA*, *trnP-UGG*, *trnH-GUG* ^a^, *trnM-CAU*, *trnL-CAA* ^a^, *trnV-GAC* ^a^, *trnI-GAU* ^a^*, *trnI-CAU* ^a^, *trnfM-CAU*, *trnA-UGC* ^a^*, *trnR-ACG* ^a^, *trnN-GUU* ^a^, *trnL-UAG*
Other genes	Translational initiation factor gene	*infA*
	Maturase K gene	*matK*
	Subunit of the Acetyl-CoA-carboxylase gene	*accD*
	Envelope membrane protein gene	*cemA*
	c-type cytochrom synthesis gene	*ccsA*
	Protease gene	*clpP* **
Unkown genes	Conserved open reading frames	*ycf1*, *ycf2* ^a^, *ycf3* **, *ycf4*

Notes: ^a^ represents duplicate copy genes; * and ** represent one intron and two introns in protein-coding genes, respectively.

**Table 3:** The intron types of *Dendrobium linawianum* chloroplast genes.

Gene	Location	Start Codon	Stop Codon	Exon I/bp	Intron I/bp	Exon II/bp	Intron II/bp	Exon III/bp
trnK-UUU	LSC	GGG	CCA	37	2781	35		
rps16	LSC	ATG	TAA	40	891	248		
trnG-GCC	LSC	GCG	GCT	31	671	59		
atpF	LSC	ATG	TAG	145	939	410		
rpoC1	LSC	ATG	TAG	432	761	1608		
ycf3	LSC	ATG	TAA	124	721	230	745	153
trnL-UAA	LSC	GGG	CCA	35	793	50		
trnV-UAC	LSC	AGG	CTA	39	581	37		
clpP	LSC	ATG	TAA	71	959	294	671	229
petB	LSC	ATG	TAG	6	728	642		
petD	LSC	ATG	TAA	8	860	496		
rpl16	LSC	ATG	TAG	9	1170	399		
rpl2	IR	ATA	TAG	385	663	431		
ndhB	IR	ATG	TAG	775	699	758		
trnI-GAU	IR	GGG	CCA	37	944	35		
trnA-UGC	IR	GGG	TCC	38	801	34		

Notes: LSC, Large Single-Copy; SSC, Small Single-Copy; IRA, Inverted Repeat A; IRB, Inverted Repeat B.

**Table 4:** Codon usage in the chloroplast genome of *Dendrobium linawianum*.

Amino Acid	Codon	Number	RSCU	Amino Acid	Codons	Number	RSCU
Ala	GCA	642	1.19	Pro	CCA	473	1.09
	GCC	286	0.53		CCC	386	0.89
	GCG	237	0.44		CCG	218	0.50
	GCU	986	1.83		CCU	653	1.51
Cys	UGC	158	0.60	Gln	CAA	1213	1.48
	UGU	372	1.40		CAG	425	0.52
Asp	GAC	345	0.38	Arg	AGA	854	1.88
	GAU	1479	1.62		AGG	333	0.73
Glu	GAA	1782	1.46		CGA	590	1.30
	GAG	655	0.54		CGC	149	0.33
Phe	UUC	983	0.78		CGG	203	0.45
	UUU	1546	1.22		CGU	601	1.32
Gly	GGA	1060	1.55	Ser	AGC	213	0.36
	GGC	283	0.41		AGU	700	1.18
	GGG	503	0.74		UCA	660	1.12
	GGU	889	1.30		UCC	600	1.01
His	CAC	257	0.45		UCG	328	0.55
	CAU	877	1.55		UCU	1050	1.77
Ile	AUA	1029	0.88	Thr	ACA	648	1.22
	AUC	787	0.68		ACC	378	0.71
	AUU	1676	1.44		ACG	251	0.47
Lys	AAA	1805	1.42		ACU	855	1.60
	AAG	745	0.58	Val	GUA	788	1.40
Leu	CUA	617	0.85		GUC	302	0.54
	CUC	317	0.43		GUG	373	0.66
	CUG	341	0.47		GUU	785	1.4
	CUU	911	1.25	Trp	UGG	812	1.00
	UUA	1253	1.72	Tyr	UAC	313	0.42
	UUG	942	1.29		UAU	1165	1.58
Met	AUG	1001	1.00	Terminater	UAA	114	1.08
Asn	AAC	483	0.47		UAG	106	1.01
	AAU	1575	1.53		UGA	95	0.90

## Data Availability

The original contributions presented in this study are included in the article. Further inquiries can be directed to the corresponding author.

## Supplementary Materials

The following supporting information can be downloaded at: https://www.mdpi.com/article/10.3390/cimb47100869/s1.

## References

[1] Xiang X.G., Schuiteman A., Li D.Z., Huang W.C., Chung S.W., Li J.W., Zhou H.L., Jin W.T., Lai Y.J., Li Z.Y. (2013). Molecular systematics of *Dendrobium* (Orchidaceae, Dendrobieae) from mainland Asia based on plastid and nuclear sequences. Mol. Phylogenet. Evol..

[2] Xiang X.G., Mi X.C., Zhou H.L., Li J.W., Chung S.W., Li D.Z., Huang W.C., Jin W.T., Li Z.Y., Huang L.Q. (2016). Biogeographical diversification of mainland Asian *Dendrobium* (Orchidaceae) and its implications for the historical dynamics of evergreen broad-leaved forests. J. Biogeogr..

[3] Cakova V., Bonte F., Lobstein A. (2017). *Dendrobium*: Sources of active ingredients to treat age-related pathologies. Aging Dis..

[4] Bao X.S., Shun Q.S., Chen L.Z. (2001). The Medicinal Plants of Dendrobium (Shi-hu) in China.

[5] Wood H.P. (2006). The Dendrobiums.

[6] Zhu G.H., Ji Z.H., Wood J.J., Wood H.P., Wu C.Y., Raven P.H., Hong D.Y. (2009). Dendrobium. Flora of China.

[7] Teixeirada Silva J.A., Jin X., Dobránszki J., Lu J., Wang H., Zotz G., Cardoso J.C., Zeng S. (2016). Advancesin *Dendrobium* molecular research: Applications in genetic variation, identification and breeding. Mol. Phylogenet. Evol..

[8] Wang S., Xie Y. (2004). The Red List of Chincese Species.

[9] Ding G., Zhang D.Z., Ding X.Y., Zhou Q., Zhang W.C., Li X.X. (2008). Genetic variation and conservation of the endangered Chinese endemic herb *Dendrobium officinale* based on SRAP analysis. Plant Syst. Evol..

[10] Xu X., Liu X., Ge S., Jensen J.D., Hu F.Y., Li X., Dong Y., Gutenkunst R.N., Fang L., Huang L. (2012). Resequencing 50 accessions of cultivated and wild rice yields markers for identifying agronomically important genes. Nat. Biotechnol..

[11] Yukawa T., Uehara K. (1996). Vegetative diversification and radiation in subtribe Dendrobiinae (Orchidaceae): Evidence from chloroplast DNA phylogeny and anatomical characters. Plant Syst. Evol..

[12] Zhang T., Wang Z.T., Xu L.S., Zhou K.Y. (2005). Application of mitochondrial nad 1 intron 2 sequences to molecular identification of some species of *Dendrobium* Sw. Chin. Tradit. Herb. Drugs.

[13] Niu Z., Pan J., Xue Q., Zhu S., Liu W., Ding X. (2018). Plastome-wide comparison reveals new SNV resources for the authentication of *Dendrobium huoshanense* and its corresponding medicinal slice (Huoshan Fengdou). Acta Pharmacol. Sin..

[14] Yukawa T., Kita K., Handa T., Wilson K.L., Mossison D.A. (2000). DNA phylogeny and morphological diversification of Australian *Dendrobium* (Orchidaceae). Monocots: Systematics and Evolution.

[15] Clements M.A. (2003). Molecular phylogenetic systematics in the Dendrobiinae (Orchidaceae), with emphasis on *Dendrobium* section *Pedilonum*. Telopea.

[16] Clements M.A. (2006). Molecular phylogenetic systematics in Dendrobieae (Orchidaceae). Aliso.

[17] Burke J.M., Bayly M.J., Adams P.B., Ladiges P.Y. (2008). Molecular phylogenetic analysis of *Dendrobium* (Orchidaceae), with emphasis on the Australian section Dendrocoryne, and implications for generic classification. Aust. Sys.Bot..

[18] Adams P.B. (2011). Systematics of Dendrobiinae (Orchidaceae), with special reference to Australian taxa. Bot. J. Linn. Soc..

[19] Schuiteman A. (2011). *Dendrobium* (Orchidaceae): To split or not to split?. Gard. Bull. Singap..

[20] Zha X.Q., Luo J.P., Wei P. (2009). Identification and classification of *Dendrobium candidum* species by fingerprint technology with capillary electrophoresis. S. Afr. J. Bot..

[21] Yang L., Wang Z., Xu L. (2006). Simultaneous determination of phenols (bibenzyl, phenanthrene, and fluorenone). *Dendrobium* species by high-performance liquid chromatography with diode array detection. J. Chromatogr. A.

[22] Takamiya T., Wongsawad P., Sathapattayanon A., Tajima N., Suzuki S., Kitamura S., Iijima H., Yukawa T. (2014). Molecular phylogenetics and character evolution of morphologically diverse groups, *Dendrobium* section *Dendrobium* and allies. AoB Plants.

[23] Hou B.W., Luo J., Zhang Y.S., Niu Z.T., Xue Q.Y., Ding X.Y. (2017). Iteration expansion and regional evolution: Phylogeography of *Dendrobium officinale* and four related taxa in southern China. Sci. Rep..

[24] Shen J., Ding X., Liu D., Ding G., He J., Li X., Tang F., Chu B. (2006). Intersimple sequence repeats (ISSR) molecular finger printing markers for authenticating populations of *Dendrobium officinale* Kimura et Migo. Biol. Pharm. Bull..

[25] Feng S.G., Lu J.J., Gao L., Liu J.J., Wang H.Z. (2014). Molecular phylogeny analysis and species identification of *Dendrobium* (Orchidaceae) in China. Biochem. Genet..

[26] Kang J.Y., Lu J.J., Qiu S., Chen Z., Liu J.J., Wang H.Z. (2015). *Dendrobium* SSR markers play a good role in genetic diversity and phylogenetic analysis of Orchidaceae species. Sci. Hortic..

[27] Tsai C.C., Peng C.I., Huang S.C., Huang P.L., Chou C.H. (2004). Determination of the genetic relationship of *Dendrobium* species (Orchidaceae) in Taiwan based on the sequence of the internal transcribed spacer of ribosomal DNA. Sci. Hortic..

[28] Xu S.Z., Li D.Z., Li J.W., Xiang X.G., Jin W.T., Huang W.C., Jin X.H., Huang L.Q. (2015). Evaluation of the DNA barcodes in *Dendrobium* (Orchidaceae) from mainland Asia. PLoS ONE.

[29] Zhu B., Gan C.C., Wang H.C. (2021). Characteristics of the complete chloroplast genome of *Dendrobium thyrsiflorum* and its phylogenetic relationship analysis. Biotechnol. Bull..

[30] Li Z.W., Qiu Q., Lang J.Q., Wu Y.M., Du H.H., Zhou N. (2022). Sequence analysis of complete chloroplast genome of *Dendrobium heterocarpum* Lindl. and *Dendrobium trigonopus* Rchb. f. Chin. Tradit. Herb. Drugs.

[31] Shang M.Y., Wang J.L., Zhou Y., Zhang M.C., Liu Y.L., Duan B.Z. (2023). Analysis of chloroplast genome structure and phylogeny of endangered *Dendrobium devonianum*. Chin. Tradit. Herb. Drug.

[32] Wicke S., Schneeweiss G.M., de Pamphilis C.W., Muller K.F., Quandt D. (2011). The evolution of the plastid chromosome in land plants: Gene content, gene order, gene function. Plant Mol. Biol..

[33] Rodríguez-Ezpeleta N., Brinkmann H., Burey S.C., Roure B., Burger G., Löffelhardt W., Bohnert H.J., Philippe H., Lang B.F. (2005). Monophyly of primary photosynthetic eukaryotes: Green plants, red algae, and glaucophytes. Curr. Biol..

[34] Du X.Y., Zeng T., Feng Q., Hu L., Luo X., Weng Q., He J., Zhu B. (2020). The complete chloroplast genome sequence of yellow mustard (*Sinapis alba* L.) and its phylogenetic relationship to other Brassicaceae species. Gene.

[35] Lo S.F., Nalawade S.M., Mulabagal V., Matthew S., Chen C.L., Kuo C.L., Tsay H.S. (2004). In vitro propagation by asymbiotic seed germination and 1,1-diphenyl-2-picrylhydrazyl (DPPH) radical scavenging activity studies of tissue culture raised plants of three medicinally important species of *Dendrobium*. Biol. Pharm. Bull..

[36] Ma L., Chen S.Q., Zhuang L.B. (2019). Evaluation on Ornamental Characters of 35 Species of *Dendrobium*. Subtop. Plant Sci..

[37] Doyle J.J., Doyle J.L. (1987). A rapid DNA isolation procedure for small quantities of fresh leaf tissue. Phytochem. Bull..

[38] Jin J.J., Yu W.B., Yang J.B., Song Y., De Pamphilis C.W., Yi T.S., Li D.Z. (2020). GetOrganelle: A fast and versatile toolkit for accurate de novo assembly of organelle genomes. Genome Biol..

[39] Bankevich A., Nurk S., Antipov D., Gurevich A.A., Dvorkin M., Kulikov A.S., Lesin V.M., Nikolenko S.I., Pham S., Prjibelski A.D. (2012). SPAdes: A new genome assembly algorithm and its applications to single-cell sequencing. J. Comput. Biol..

[40] Shi L.C., Chen H.M., Jiang M., Wang L.Q., Wu X., Huang L.F., Liu C. (2019). CPGAVAS2, an integrated plastome sequence annotator and analyzer. Nucleic Acids Res..

[41] Lohse M., Drechsel O., Bock R. (2007). OrganellarGenomeDRAW (OGDRAW): A tool for the easy generation of high-quality custom graphical maps of plastid and mitochondrial genomes. Curr. Genet..

[42] Beier S., Thiel T., Münch T., Scholz U., Mascher M. (2017). MISA-web: A web server for microsatellite prediction. Bioinformatics.

[43] Chen C.J., Chen H., Zhang Y., Thomas H.R., Frank M.H., He Y., Xia R. (2020). TBtools: An integrative toolkit developed for interactive analyses of big biological data. Mol. Plant.

[44] Amiryousefi A., Hyvönen J., Poczai P. (2018). IRscope: An online program to visualize the junction sites of chloroplast genomes. Bioinformatics.

[45] Katoh K., Standley D.M. (2013). MAFFT multiple sequence alignment software version 7: Improvements in performance and usability. Mol. Biol. Evol..

[46] Ronquist F., Teslenko M., Van Der Mark P., Ayres D.L., Darling A., Höhna S., Larget B., Liu L., Suchard M.A., Huelsenbeck J. (2012). MrBayes 3.2: Efficient Bayesian phylogenetic inference and model choice across a large model space. Syst. Biol..

[47] Tamura K., Stecher G., Kumar S. (2021). MEGA11: Molecular Evolutionary Genetics Analysis Version 11. Mol. Biol. Evol..

[48] Daniell H., Lin C.S., Yu M., Chang W.J. (2016). Chloroplast genomes: Diversity, evolution, and applications in genetic engineering. Genome Biol..

[49] Kim K.J., Lee H.L. (2004). Complete chloroplast genome sequences from Korean ginseng (*Panax* schinseng nees) and comparative analysis of sequence evolution among 17 vascular plants. DNA Res..

[50] Park S., An B., Park S. (2018). Reconfiguration of the plastid genome in *Lamprocapnos spectabilis*: IR boundary shifting, inversion, and intraspecific variation. Sci. Rep..

[51] Wang W.C., Chen S.Y., Zhang X.Z. (2018). Whole-genome comparison reveals divergent IR borders and mutation hotspots in chloroplast genomes of herbaceous bamboos (bambusoideae: Olyreae). Molecules.

[52] Mukhopadhyay P., Basak S., Ghosh T.C. (2007). Nature of selective constraints on synonymous codon usage of rice differs in GC-poor and GC-rich genes. Gene.

[53] Gao Y.X., Zhou Y.Y., Xie Y., Feng L., Shen S.G. (2018). The complete chloroplast genome sequence of an endangered Orchidaceae species *Dendrobium monilforme* and its phylogenetic implications. Conserv. Genet. Resour..

[54] Zhu A., Guo W., Gupta S., Fan W., Mower J.P. (2016). Evolutionary dynamics of the plastid inverted repeat: The effects of expansion, contraction, and loss on substitution rates. New Phytol..

[55] Saha M.C., Cooper J.D., Mian M.A., Chekhovskiy K., May G.D. (2006). Tall fescue genomic SSR markers: Development and transferability across multiple grass species. Theor. Appl. Genet..

[56] Curci P.L., De Paola D., Danzi D., Vendramin G.G., Sonnante G. (2015). Complete chloroplast genome of the multifunctional crop globe artichoke and comparison with other Asteraceae. PLoS ONE.

[57] Yap J.Y., Rohner T., Greenfield A., Van Der Merwe M., McPherson H., Glenn W., Kornfeld G., Marendy E., Pan A.Y., Wilton A. (2015). Complete chloroplast genome of the Wollemi pine (*Wollemia nobilis*): Structure and evolution. PLoS ONE.

[58] Park I., Kim W.J., Yeo S.M., Choi G., Kang Y.M., Piao R.Z., Moon B.C. (2017). The Complete Chloroplast Genome Sequences of *Fritillaria ussuriensis* Maxim. and *Fritillaria cirrhosa* D. Don, and Comparative Analysis with Other *Fritillaria* Species. Molecules.

[59] Wolfe K.H., Li W.H., Sharp P.M. (1987). Rates of nucleotide substitution vary greatly among plant mitochondrial, chloroplast, and nuclear DNAs. Proc. Natl. Acad. Sci. USA.

[60] Plotkin J., Kudla G. (2011). Synonymous but not the same: The causes and consequences of codon bias. Nat. Rev. Genet..

[61] Huang H., Shi C., Liu Y., Mao S.Y., Gao L.Z. (2014). Thirteen *Camellia* chloroplast genome sequences determined by high-throughput sequencing: Genome structure and phylogenetic relationships. BMC Evol. Biol..

[62] Raubeson L.A., Peery R., Chumley T.W., Dziubek C., Fourcade H.M., Boore J.L., Jansen R.K. (2007). Comparative chloroplast genomics: Analyses including new sequences from the angiosperms *Nuphar advena* and *Ranunculus macranthus*. BMC Genom..

[63] Chang C.C., Lin H.C., Lin I.P., Chow T.Y., Chen H.H., Chen W.H., Cheng C.H., Lin C.Y., Liu S.M., Chang C.C. (2006). The chloroplast genome of *Phalaenopsis aphrodite* (Orchidaceae): Comparative analysis of evolutionary rate with that of grasses and its phylogenetic implications. Mol. Biol. Evol..

[64] Wang B., Qiu Y.L. (2006). Phylogenetic distribution and evolution of mycorrhizas in land plants. Mycorrhiza.

[65] Martín M., Sabater B. (2010). Plastid *ndh* genes in plant evolution. Plant Physiol. Biochem..

[66] Selosse M.A., Albert B., Godelle B. (2001). Reducing the genome size of organelles favours gene transfer to the nucleus. Trends Ecol. Evol..

[67] Adams K.L., Palmer J.D. (2003). Evolution of mitochondrial gene content: Gene loss and transfer to the nucleus. Mol. Phylogenet. Evol..

[68] Gomez S.M., Brown M.J.M., Pironon S., Bureš P., Verde Arregoitia L.D., Veselý P., Elliott T.L., Zedek F., Pellicer J., Forest F. (2024). Genome size is positively correlated with extinction risk in herbaceous angiosperms. New Phytol..

[69] Yang J.B., Tang M., Li H.T., Zhang Z.R., Li D.Z. (2013). Complete chloroplast genome of the genus *Cymbidium*: Lights into the species identification, phylogenetic implications and population genetic analyses. BMC Evol. Biol..

[70] Hu H., Hu Q., Al-Shehbaz I.A., Luo X., Zeng T., Guo X., Liu J. (2016). Species delimitation and interspecific relationships of the genus *Orychophragmus* (Brassicaceae) inferred from whole chloroplast genomes. Front. Plant Sci..

[71] Du Y.P., Bi Y., Yang F.P., Zhang M.F., Chen X.Q., Xue J., Zhang X.H. (2017). Complete chloroplast genome sequences of *Lilium*: Insights into evolutionary dynamics and phylogenetic analyses. Sci. Rep..

[72] Yu X.Q., Drew B.T., Yang J.B., Gao L.M., Li D.Z. (2017). Comparative chloroplast genomes of eleven *Schima* (Theaceae) species: Insights into DNA barcoding and phylogeny. PLoS ONE.

[73] Zhu S.Y., Niu Z.T., Xue Q.Y., Wang H., Xie X.Z., Ding X.Y. (2018). Accurate authentication of *Dendrobium officinale* and its closely related species by comparative analysis of complete plastomes. Acta Pharm. Sin. B.

[74] Kim Y.K., Jo S., Cheon S.H., Kwak M., Kim Y.D., Kim K.J. (2020). Plastome evolution and phylogeny of subtribe Aeridinae (Vandeae, Orchidaceae). Mol. Phylogenet. Evol..

[75] Liu D.K., Tu X.D., Zhao Z., Zeng M.Y., Zhang S., Ma L., Zhang G.Q., Wang M.M., Liu Z.J., Lan S.R. (2020). Plastid phylogenomic data yield new and robust insights into the phylogeny of *Cleisostoma*-*Gastrochilus* clades (Orchidaceae, Aeridinae). Mol. Phylogenet. Evol..

