# Supplementary material for: Determination of the Phylogenetic Relationship of Dendrobium linawianum (Orchidaceae) Based on Comparative Analysis of Complete Chloroplast Genomes
Source: Curr Issues Mol Biol. 2025 Oct 21;47(10):869. doi: 10.3390/cimb47100869 (PMC12563224; doi:10.3390/cimb47100869)
Supplement: Supplementary file 1 [file cimb-47-00869-s001.zip › cimb-3886075-supplementary.docx]

**Supporting information**

**Table S1.** Characteristics of the chloroplast genomes for 35 *Dendrobium* species

| Species | Accession Number | Total length (bp) | LSC (bp) | IR(bp) | SSC (bp) | AT Content (%) |
| --- | --- | --- | --- | --- | --- | --- |
| *D*. *aduncum* | LC348859 | 152,104 | 84,944 | 26,319 | 14,522 | 62.5 |
| *D*. *aphyllum* | LC490671 | 152,462 | 84,837 | 27,040 | 13,545 | 62.5 |
| *D*. *brymerianum* | LC192954 | 152,931 | 84,880 | 26,868 | 14,315 | 62.5 |
| *D*. *cariniferum* | NC080348 | 159,552 | 86,986 | 27,028 | 18,510 | 62.9 |
| *D*. *christyanum* | MZ241112 | 157,972 | 87,717 | 25,971 | 18,313 | 62.8 |
| *D*. *chrysotoxum* | LC193517 | 151,731 | 84,785 | 26,295 | 14,356 | 62.4 |
| *D*. *chrysanthum* | LC490684 | 153,030 | 84,945 | 27,030 | 14,025 | 62.5 |
| *D*. *crepidatum* | LC490675 | 153,451 | 84,933 | 27,030 | 14,458 | 62.5 |
| *D. crystallinum* | LC490678 | 152,865 | 85,015 | 26,890 | 14,070 | 62.5 |
| *D. ellipsophyllum* | LC193519 | 152,026 | 84,930 | 26,304 | 14,488 | 62.5 |
| *D*. *fimbriatum* | LC193521 | 151,673 | 84,763 | 26,291 | 14,328 | 62.4 |
| *D*. *exile* | LC193522 | 151,294 | 84,363 | 26,308 | 14,315 | 62.3 |
| *D*. *flexicaule* | LC348965 | 152,191 | 84,921 | 26,301 | 14,455 | 62.5 |
| *D*. *gratiosissimum* | LC490662 | 151,803 | 84,908 | 26,308 | 14,279 | 62.5 |
| *D*. *henryi* | LC193513 | 151,850 | 84,878 | 26,303 | 14,366 | 62.4 |
| *D*. *hercoglossum* | LC490402 | 152,196 | 84,994 | 26,336 | 14,530 | 62.5 |
| *D*. *jenkinsii* | LC193515 | 151,717 | 84,734 | 26,285 | 14,413 | 62.5 |
| *D*. *loddigesii* | LC490673 | 152,384 | 84,756 | 27,027 | 13,574 | 62.5 |
| *D*. *lohohense* | LC490670 | 153,202 | 84,932 | 27,036 | 14,198 | 62.5 |
| *D*. *longicornu* | MN227146 | 160,024 | 88,075 | 25,403 | 21,143 | 62.9 |
| *D. linawianum* | NC087858 | 150,497 | 84,771 | 25,969 | 13,788 | 62.4 |
| *D*. *moniliforme* | LC490386 | 150,754 | 84,818 | 25,906 | 14,124 | 62.5 |
| *D*. *moschatum* | OM161978 | 159,701 | 87,635 | 27,261 | 17,544 | 62.7 |
| *D*. *nobile* | KX377961 | 152,018 | 84,944 | 26,285 | 14,504 | 62.5 |
| *D*. *officinale* | MN617017 | 152,041 | 83,708 | 26,268 | 14,413 | 62.5 |
| *D*. *parishii* | LC193518 | 151,689 | 84,703 | 26,295 | 14,396 | 62.4 |
| *D*. *pendulum* | LC490663 | 153,248 | 85,852 | 26,398 | 14,600 | 62.5 |
| *D*. *primulinum* | LC490397 | 150,767 | 84,442 | 26,175 | 13,975 | 62.5 |
| *D*. [*spatella*](http://www.iplant.cn/info/Dendrobium%20spatella) | LC193511 | 151,829 | 84,794 | 26,308 | 14,419 | 62.4 |
| *D*. *stuposum* | LC715249 | 151,513 | 84,564 | 26,268 | 14,413 | 62.4 |
| *D*. *strongylanthum* | LC490672 | 152,888 | 84,918 | 27,022 | 13,926 | 62.3 |
| *D*. *terminale* | NC071775 | 151,490 | 84,451 | 26,305 | 14,432 | 62.5 |
| *D*. *thyrsiflorum* | NC047439 | 151,686 | 84,749 | 26,293 | 14,315 | 62.5 |
| *D*. *wardianum* | LC490666 | 153,627 | 84,994 | 27,048 | 14,537 | 62.5 |
| *D*. *wattii* | NC045856 | 159,366 | 87,192 | 26,876 | 18,422 | 62.8 |

Notes: LSC, large single-copy; SSC, small single-copy; IRA, inverted repeat A; IRB, inverted repeat B


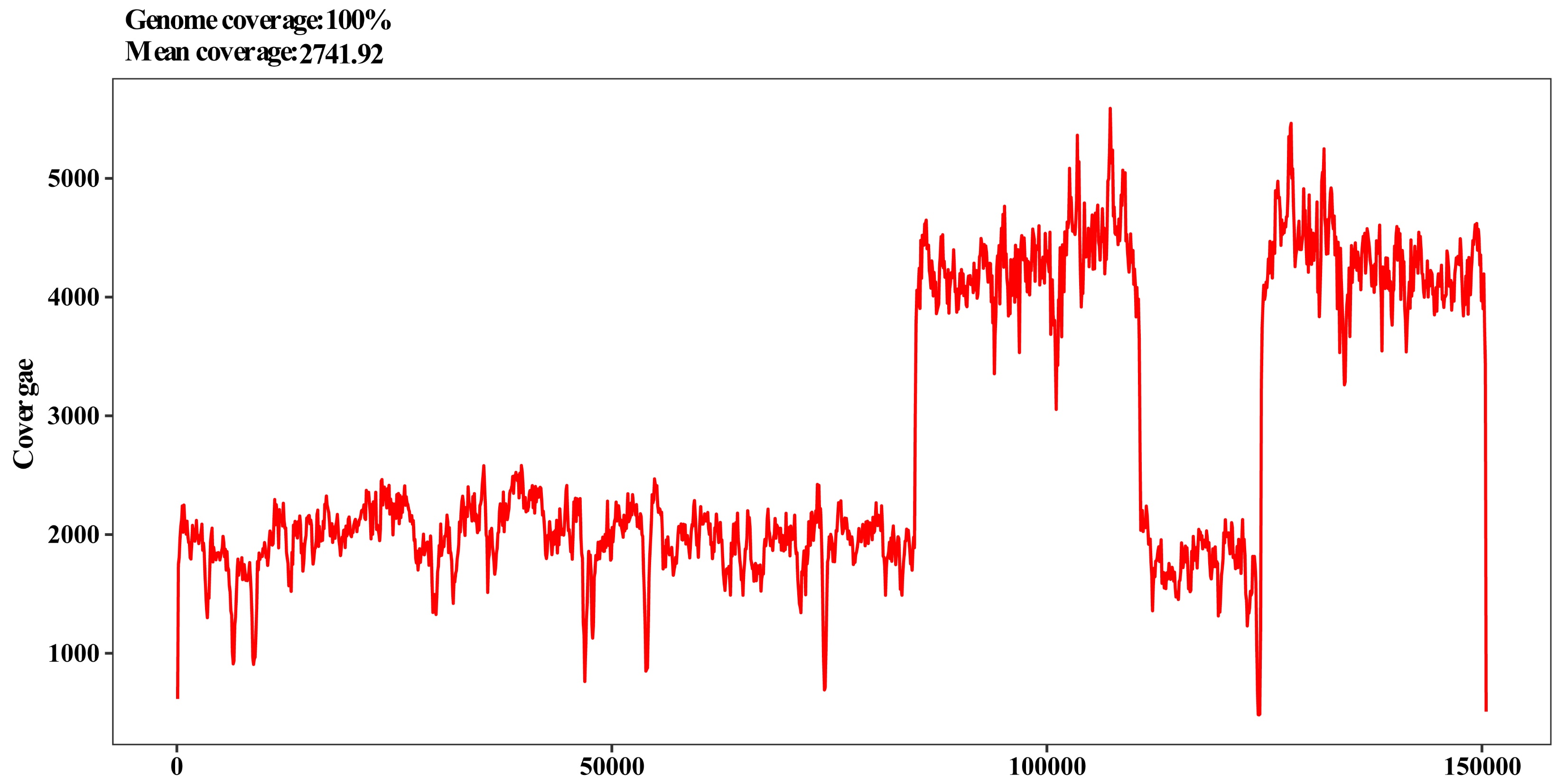


**Figure S1.** The average coverage depth and genome coverage.
